# Colposcopy attendance and deprivation: A retrospective analysis of 27 193 women in the NHS Cervical Screening Programme

**DOI:** 10.1038/bjc.2015.176

**Published:** 2015-05-21

**Authors:** E Douglas, J Wardle, N J Massat, J Waller

**Affiliations:** 1 Health Behaviour Research Centre, Department of Epidemiology & Public Health, University College London, Gower Street, London WC1E 6BT, UK; 2 Centre for Cancer Prevention, Wolfson Institute of Preventive Medicine, Queen Mary University of London, London, UK

**Keywords:** cancer screening, colposcopy, socioeconomic inequalities

## Abstract

**Background:**

Attendance for cervical screening is socially graded, but little is known about patterns of attendance for colposcopy following an abnormal screening result.

**Methods:**

Logistic regression was used to regress colposcopy attendance status for 27 193 women against age and area-level deprivation, adjusting for ethnicity.

**Results:**

Colposcopy attendance was high at 8 weeks (89%) and 4 months post-referral (94%) but women living in the most deprived areas were significantly less likely to attend.

**Conclusions:**

The high overall attendance rates at colposcopy are encouraging but lower attendance among women in the most income-deprived areas indicates that even when these women attend primary cervical screening, they remain at higher risk of missing out on the benefits of the programme.

Introduction of an organised cervical screening programme in the UK in 1988 dramatically reduced cervical cancer incidence and mortality (Quinn *et al*, 1999; Peto *et al*, 2004), and protected the population against rises in incidence that would probably have occurred because of changes in sexual behaviour (Mercer *et al*, 2013). The cervical screening programme offers 3–5 yearly testing for cytological abnormalities (Health and Social Care Information Centre, 2013), with referral to colposcopy for further investigation and treatment if abnormalities are detected. The success of the programme depends on high attendance at both primary screening and colposcopy.

Screening attendance has consistently been found to be lower in women who live in more deprived areas (Baker and Middleton, 2003; Webb *et al*, 2004; Bang *et al*, 2012), have lower levels of education (Sabates and Feinstein, 2006) or lower socioeconomic status (SES) (Moser *et al*, 2009). Other factors such as younger age (Lancucki *et al*, 2010; Albrow *et al*, 2012) and non-white ethnicity (Webb *et al*, 2004; Moser *et al*, 2009) are strong predictors of lower attendance although ethnicity may be confounded with SES. Much less is known about patterns of attendance at colposcopy follow-up. In the TOMBOLA trial, colposcopy attendance was very high (around 93%) (Sharp *et al*, 2012). However, attendance was lower in women who were younger and less educated. Late attendance for colposcopy (more than 6 months after the original appointment) was also associated with having less education, and it predicted non-attendance for subsequent colposcopy appointments (Sharp *et al*, 2012).

We know of no analyses of socioeconomic patterns of attendance at colposcopy using individual-level attendance data from the national screening programme. National appointment-level data for England showed attendance of 77% in 2012–2013; but this underestimates attendance at the individual level, because it fails to account for women who miss or cancel one appointment but attend a second one soon afterwards (Health and Social Care Information Centre, 2013). Appointment-level data may mask demographic patterns of attendance if certain groups are disproportionately likely to rearrange but subsequently attend appointments.

We used patient-level data, which is now available in some areas in England (NHS Cancer Screening Programme, 2011), to explore demographic differences in colposcopy attendance. We hypothesised that patterns of attendance would mirror those in primary screening, with women of lower SES less likely to attend.

## Materials and Methods

### Data and variables

The source of data was the East of England Cyres Colposcopy database, covering a screening eligible population of ∼1.5 million women (ONS, 2014a). Anonymised data were extracted for all women referred to colposcopy following an abnormal screening result from 2006–2013. Referral date is defined as the date at which cytology is reported. Colposcopy attendance was ascertained by tracking patients from referral to appointment status 8 weeks later, allowing them to re-book their initial appointment within that time period. Women were categorised as ‘attenders' or ‘non-attenders' at 8 weeks. This time interval was chosen because 98% of women referred to colposcopy are offered an appointment within 8 weeks (Health and Social Care Information Centre, 2013). A secondary analysis examined attendance at 4 months. This interval was chosen because cervical cancer detected <4 months after a screening referral is considered to be ‘screen-detected' (NHS Cancer Screening Programme, 2011).

For each individual, data were downloaded on age and Lower Super Output Area (LSOA) for the postcode of their home address at the time of referral. We used LSOA to access local area-level values for deprivation (the income domain of the Index of Multiple Deprivation) (Department for Communities and Local Government, 2011) and ethnic diversity (percentage of the population from white ethnic backgrounds) (ONS, 2014b). The income domain was chosen because it is likely to be relatively homogeneous within LSOAs and so is more likely to reflect individual-level income (ONS, 2007).

Categorical variables were constructed for age (25–34, 35–44 and 45–64 years) and deprivation (quintiles based on national data (Knowledge & Information Team, Public Health England, 2011)). Ethnic diversity was used as a continuous variable.

The study was exempt from the need for ethical approval under the UCL Ethics Committee guidelines.

### Analysis

Multiple logistic regression (Hosmer and Lemeshow, 2004) was used to regress colposcopy attendance status (using 8-week and 4-month cut-offs) against age and deprivation, prior to and after adjusting for ethnicity.

## Results

### Characteristics of the sample

During 2006–2013, 27 193 women were referred for colposcopy. Where an individual woman had more than one colposcopy referral, we only included the first. Women had a mean age of 35 years (standard deviation=9.1), mostly lived in areas within the upper four quintiles of deprivation, reflecting the relative affluence of the East of England in comparison with the country as a whole, and came from areas of predominantly white ethnicity.

### Colposcopy attendance and socio-demographic variables

Overall, 89.3% of women attended for colposcopy within 8 weeks (Table 1). In unadjusted analyses, women in the lowest quintile of income had significantly lower odds of attendance compared with the highest income quintile (86.6% compared with 89.1%, odds ratio (OR)=0.79, 95% CI: 0.68–0.91). There was no significant association between age and attendance within 8 weeks. In the model adjusted for area-level ethnic diversity, the OR for the lowest income group was slightly attenuated but remained significant (OR=0.83, 95% CI: 0.72–0.97).

When we examined attendance within 4 months, mean attendance was 94% (Table 2). In the unadjusted analysis, women living in the lowest income area were significantly less likely to attend (92.5% *vs* 94.1% OR=0.76, 95% CI: 0.63–0.93). In the adjusted model, women living in the lowest quintile remained significantly less likely to attend, though as before, the association was slightly attenuated after adjustment for area-level ethnicity (OR=0.81, 95% CI: 0.67–0.98). Women aged 45–64 years were significantly more likely to attend colposcopy in unadjusted (OR=1.25, 95% CI: 1.08–1.45) and adjusted models (OR=1.23, 95% CI: 1.06–1.43), than women in the 25–34 year reference category.

## Discussion

This is the first study of individual-level colposcopy attendance following an abnormal screening result in the organised cervical screening programme in England. We investigated attendance within 8 weeks, which is the value used in appointment-level statistics, and attendance within 4 months to include ‘late attenders' who may still attend within the time frame in which cervical cancer, if diagnosed, is considered to be ‘screen-detected' (NHS Cancer Screening Programme, 2011).

Attendance within 8 weeks of referral was lower than attendance in the multi-centre population-based randomised controlled trial nested in the NHS Cervical Screening Programme (TOMBOLA) (Sharp *et al*, 2012) (89% compared with 93%) but our 4-month attendance was similar (94%). There is a fail-safe process in place in England to manage women who do not attend colposcopy to minimise loss to follow-up. This includes sending reminder letters and informing the GP of non-attendance, but may vary between colposcopy clinics (NHS Cancer Screening Programme, 2010). The higher attendance at 4 months may be in part due to efforts to encourage women to attend over the extended period; efforts that appear to be effective across all quintiles of deprivation.

High levels of attendance (88%) have also been reported for referral to colonoscopy following a positive faecal occult blood test in the colorectal cancer screening programme (Morris *et al*, 2012). These findings suggest that once a cancer screening invitation is accepted, compliance with recommended follow-up and treatment is likely to be high. That said, minimising missed appointments, which increase the risk of delayed diagnosis of cervical cancer (NHS Cancer Screening Programme, 2011) and are costly to the NHS (Bech, 2005), remains important despite overall high rates of attendance.

Women from the most income-deprived areas had lower colposcopy attendance at both time points, even after adjusting for area-level ethnicity, but the dose–response association often observed between screening uptake and SES was not seen. This suggests that barriers to colposcopy attendance may be concentrated in the most deprived groups, although this requires further exploration. The association between deprivation and low uptake is consistent with findings from two retrospective studies of colposcopy clinic data in England (Sanders *et al*, 1992; Orbell *et al*, 2006). The TOMBOLA study also found that attendance was lower in women with no post-school education (Sharp *et al*, 2012).

Older women (aged 45–64 years) were significantly more likely to attend colposcopy within 4 months than younger women, consistent with the TOMBOLA study (Sharp *et al*, 2012). The significant association between age and colposcopy attendance was, however, not found at 8 weeks. This suggests that of the women who have not attended at 8 weeks, older women are more likely to have delayed attendance but attended within 4 months. Analyses of age differences in primary cervical screening attendance suggest that older women are less likely to cite difficulties in either making an appointment or finding time to attend screening, but may have a lower perceived risk of cervical cancer (Waller *et al*, 2011), which may lead to delayed attendance. ‘Late attenders' at first referral to colposcopy have been found to be more likely to not attend subsequent follow-up colposcopy appointments (Sharp *et al*, 2012), therefore gaining further understanding of this issue is an important avenue for future research.

Explanations for non-attendance at colposcopy include physical (Marteau *et al*, 1990), psychological (Marteau *et al*, 1990; Wardle *et al*, 1995; McCaffery *et al*, 2006; Gray *et al*, 2006), educational (Lindau *et al*, 2006) and practical factors (Orbell *et al*, 2006; Balasubramani *et al*, 2008; Linsell *et al*, 2010). One study found evidence that history of domestic violence is a strong predictor of colposcopy default and loss to follow-up (Collier and Quinlivan, 2014), but there is limited research on socio-demographic variation in barriers to colposcopy. Further research in this area is warranted.

This study benefited from the use of a very large sample of women from the NHS screening programme. However, using area-level variables for SES and ethnicity is a potential weakness of the study. Socioeconomic status and ethnicity data are not routinely collected by the cervical screening programme, but we were able to match to LSOA level. Lower Super Output Areas are small, homogenous geographical areas designed for neighbourhood statistical analyses (ONS, 2007). The East of England region has relatively high colposcopy attendance in comparison with other regions (Health and Social Care Information Centre, 2013), but linking the area-level measures to national quintiles may increase the relevance of these results for other regions in England. We were not able to look at mediators of the demographic patterns observed, and future work might usefully investigate the relationship between deprivation and other factors such as practical barriers and psychological well-being.

## Conclusion

The high attendance rate at colposcopy is encouraging because it indicates that, in this area of England at least, women who accept an invitation to cervical screening are likely to accept a referral to colposcopy. However, lower attendance among women in the most income-deprived areas is of concern because this suggests that even when they attend cervical screening, they are at increased risk of missing out on the benefits of the programme. There is a need for research designed to understand the mechanisms through which deprivation is linked to lower colposcopy attendance to inform future intervention development.

## Figures and Tables

**Table 1:** Analyses of variables associated with colposcopy attendance within 8 weeks of referral

			**Unadjusted models**		**Adjusted model**^a^	
	**Sample column % (*****n***)	**8 week attenders row % (*****n***)	**OR (95% CI)**	***P*****-value**	**OR (95% CI)**	***P*****-value**
	100 (27 193)	89.3 (24 294)				
**Income quintile**						
Q1—Low income	8.5 (2305)	86.6 (1996)	0.79 (0.68–0.91)	0.001*	0.83 (0.72–0.97)	0.016*
Q2	22.3 (6064)	89.6 (5434)	1.05 (0.94–1.19)	0.400	1.12 (0.99–1.26)	0.075
Q3	26.7 (7255)	89.4 (6486)	1.03 (0.92–1.15)	0.622	1.04 (0.93–1.16)	0.514
Q4	22.5 (6115)	90.2 (5517)	1.13 (1.00–1.27)	0.053	1.12 (0.99–1.26)	0.075
Q5—High income	20.1 (5454)	89.1 (4861)	1.00	—	1.00	—
**Age**						
25–34 years	55.0 (14949)	89.5 (13372)	1.00	—	1.00	—
35–44 years	27.7 (7539)	88.8 (6691)	0.93 (0.85–1.02)	0.111	0.92 (0.84–1.00)	0.053
45–64 years	17.3 (4705)	89.9 (4231)	1.05 (0.95–1.17)	0.353	1.03 (0.92–1.15)	0.607

Abbreviations: CI=confidence interval; OR=odds ratio. *P<0.05.

a Adjusted for area-level ethnic diversity (as a continuous variable).

**Table 2:** Analyses of variables associated with colposcopy attendance within 4 months of referral

			**Unadjusted models**		**Adjusted model**^a^	
	**Sample column % (*****n***)	**4 month attenders row % (*****n***)	**OR (95% CI)**	***P*****-value**	**OR (95% CI)**	***P*****-value**
	100 (27 193)	94.1 (25 594)				
**Income quintile**						
Q1—Low income	8.5 (2305)	92.5 (2131)	0.76 (0.63–0.93)	0.006*	0.81 (0.67–0.98)	0.031*
Q2	22.3 (6064)	94.5 (5731)	1.08 (0.92–1.26)	0.362	1.13 (0.97–1.33)	0.122
Q3	26.7 (7255)	94.1 (6828)	1.00 (0.86–1.16)	1.000	1.01 (0.87–1.18)	0.875
Q4	22.5 (6115)	94.4 (5771)	1.05 (0.89–1.23)	0.549	1.04 (0.89–1.22)	0.593
Q5—High income	20.1 (5454)	94.1 (5133)	1.00	—	1.00	—
**Age**						
25–34 years	55.0 (14949)	93.9 (14038)	1.00	—	1.00	—
35–44 years	27.7 (7539)	94.0 (7083)	1.01 (0.90–1.13)	0.893	1.00 (0.89–1.12)	0.941
45–64 years	17.3 (4705)	95.1 (4473)	1.25 (1.08–1.45)	0.003*	1.23 (1.06–1.43)	0.006*

Abbreviations: CI=confidence interval; OR=odds ratio. *P<0.05.

a Adjusted area-level ethnic diversity (as continuous variables).
